# Identifying stochastic dynamics from non-sequential data (DyNoSeD)

**DOI:** 10.1063/5.0314136

**Published:** 2026-02-01

**Authors:** Zhixin Lu, Łukasz Kuśmierz, Stefan Mihalas

**Affiliations:** 1 Allen Institute, 615 Westlake Ave. N, Seattle, Washington 98109, USA; 2 Department of Applied Mathematics, University of Washington, Seattle, Washington 98195, USA

## Abstract

Inferring stochastic dynamics from data is central; yet, in many applications, only unordered, non-sequential measurements are available—often restricted to limited regions of state space—so standard time-series methods fail. We introduce *DyNoSeD* (Identifying Dynamics from Non-Sequential Data), a first-principles framework that identifies unknown dynamical parameters from such non-sequential data by minimizing Fokker–Planck residuals. We develop two complementary routes: a *local* route that handles region-restricted data via local score estimation, and a *global* route that fits dynamics from globally sampled data using a kernel Stein discrepancy without density- or score estimation. When the dynamics are affine-in-the-unknown-parameters (while remaining nonlinear-in-the-state), we prove necessary-and-sufficient conditions for the *existence and uniqueness* of the inferred parameter vector and derive a sensitivity analysis that identifies which parameters are tightly constrained by the data and which remain effectively free under over-parameterization. For general non-affine parameterizations, both routes define differentiable losses amenable to gradient-based optimization. As demonstrations, we recover (i) the three parameters of a stochastic Lorenz system from non-sequential observations (region-restricted data for the local route and full steady-state data for the global route) and (ii) a 3 × 7 interaction matrix of a nonlinear gene-regulatory network derived from a published B-cell differentiation model, using only unordered steady-state samples and applying the global route. Overall, DyNoSeD provides two first-principles routes for system identification from non-sequential data, grounded in the Fokker–Planck equation, that link data, density, and stochastic dynamics.

Many stochastic systems in science are observed only through unordered “snapshots,” not time-resolved trajectories, making standard system-identification methods inapplicable. We introduce DyNoSeD (Identifying Dynamics from Non-Sequential Data), a first-principles framework that infers stochastic dynamics from non-sequential data by minimizing Fokker–Planck residuals, which connect steady-state observations to the underlying drift. DyNoSeD offers two complementary routes: a local approach tailored to region-restricted measurements via local score estimation and a global route that maps data directly to dynamics using a kernel Stein discrepancy—avoiding data-hungry density or score estimation. For both routes, we establish conditions guaranteeing existence and uniqueness of the inferred parameters and quantify which parameters are truly constrained by the available data. Together, these results provide a practical way to identify dynamics from limited steady-state data, whether measurements are confined to a region of state space or globally available but sparse.

## I. INTRODUCTION

Inferring governing dynamics from data is a central problem in science and engineering, known broadly as *system-identification*.^1-6^ When full time series are available, a variety of approaches—ranging from classical parametric identification^1^ to modern sparse-regression frameworks, such as sparse identification of nonlinear dynamics (SINDy)^3^—enable the estimation of governing equations directly from observations. Recent developments extend these ideas to high-dimensional, nonlinear, and partially observed systems using machine learning and neural differential equations.^7-9^

When continual measurements are infeasible, one may instead leverage *cross-sectional* data collected at distinct time points. Some approaches construct pseudo-time series by linking samples across time points,^10^ while more recent work casts the problem as dynamical optimal transport over Wasserstein geodesics.^11,12^ Related efforts derive estimators from the Fokker–Planck or probability-flow ordinary differential equation (ODE) perspectives for such cross-sectional settings.^8,13,14^

Here, we study a more challenging and practically common regime in which *temporal information is absent*. Data consist only of unordered *steady-state measurements*^15^ collected after the system has reached a (possibly nonequilibrium) stationary distribution. For such problems, standard time-series methods are inapplicable, and naïve attempts to recover dynamics from the stationary density are typically underdetermined: many different drifts can induce the same steady law (e.g., by adding divergence-free probability currents). A central question is, therefore, under what conditions non-sequential steady-state data suffice to identify the underlying stochastic dynamics.

In practice, non-sequential data are often available in two distinct regimes. In some experiments, measurements can be densely curated in selected regions of state space (e.g., certain experimentally accessible ranges), but are unavailable elsewhere; here, global density estimation is impossible, while local behavior is well constrained. In other settings, data are sampled unbiasedly across state space but are too sparse to support reliable global density or score estimation without imposing strong modeling biases. Our goal is to learn the dynamical parameters in *both* regimes from the same first-principles starting point and to make explicit when the resulting system-identification problem is well posed.

**FIG. 1.**
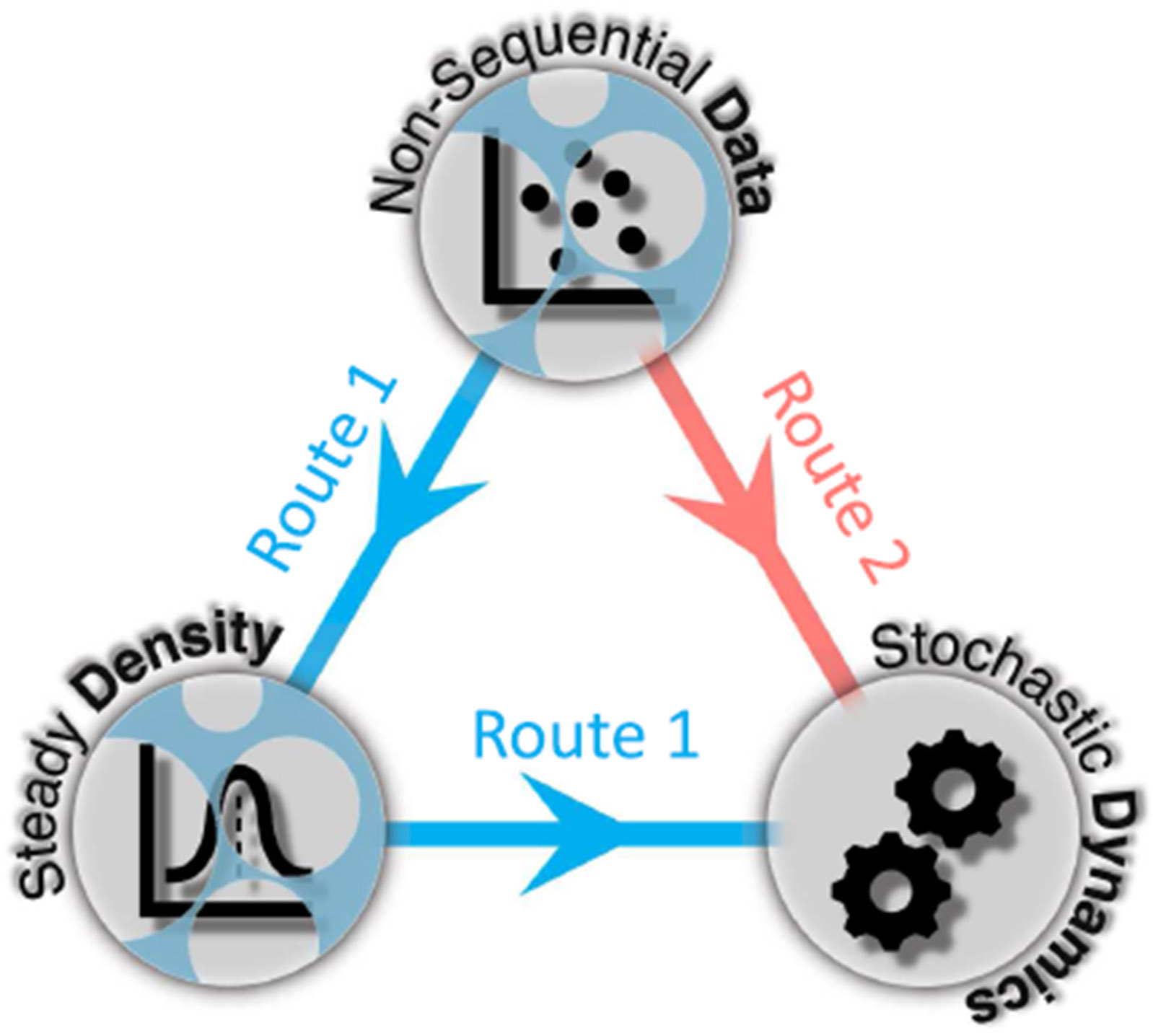
Schematic of the DyNoSeD framework. DyNoSeD links *non-sequential steady-state samples*, *steady-state distributions*, and *stochastic dynamics* through Fokker–Planck residuals (FPRs). **Route 1 (score-based; blue):** infer dynamical parameters from unordered steady-state samples—including region-restricted sampling—by estimating scores locally at a set of probe points. **Route 2 (kernel Stein discrepancy; red):** infer parameters directly from broadly distributed steady-state samples *without* density or score estimation, using a kernel Stein discrepancy derived from the same FPRs. For both routes, we provide a linear identifiability condition and a first-order sensitivity analysis when the drift is affine in the parameters.

We tackle this problem with a first-principles framework, DyNoSeD (Identifying Dynamics from Non-Sequential Data), grounded in the Fokker–Planck (FP) equation. From the FP residual, we derive two complementary learning routes tailored to these two regimes (the blue and red arrows in Fig. 1):

- **Local route (score-based; blue).** When data can be densely curated in restricted regions, we infer the dynamical parameters by minimizing the Fokker–Planck residuals (FPRs) using locally estimated scores $s(x)=\nabla_{x}\;\log\;p(x)$ at probe locations (e.g., simple kernel estimations, score matching,^16^ or the sliced score matching^17^ that is efficient for high-dimensional data). This route never requires reconstructing the global density; it only needs an accurate local structure where data are abundant.
- **Global route (Stein-based; red).** When samples are broadly distributed but not dense enough to reliably estimate a global density or score, we avoid density/score estimation altogether and instead minimize the same FP residual in a global sense via a kernel Stein discrepancy (KSD). Here, the kernel is used to define a universal reproducing-kernel Hilbert space whose test functions collectively enforce the vanishing of the residual. Using random Fourier features, we obtain a linear complexity KSD estimator that fits dynamical parameters directly from data without any explicit density or score model.

The DyNoSeD framework allows us to derive an explicit condition under which the unknown parameters can be uniquely determined from the available data. Specifically, when the prior dynamics are *affine* in their unknown parameters θ (while remaining nonlinear in the state x), both routes share a common algebraic structure: minimizing the FPRs yields a linear system, Aθ=b, evaluated at probe points (local route) or via global averages (KSD route). Beyond identifiability, we also derive a parameter-wise sensitivity analysis for the affine case that reveals which components of θ are tightly constrained by the data and which directions remain effectively free under over-parameterization. When the dynamics are not affine in θ, both routes naturally define differentiable loss functions amenable to gradient-based optimization (e.g., with automatic differentiation), while retaining the advantages of local score estimation or linear complexity KSD evaluation.

We illustrate DyNoSeD on two canonical yet challenging systems. For a stochastic Lorenz stochastic differential equation (SDE), we recover its three parameters from non-sequential data (region-restricted data via the local route and globally sampled steady-state data via the global route). For a nonlinear gene-regulatory network derived from a B-cell differentiation model,^18^ we infer the 3 × 7 interaction matrix from unordered steady-state samples using the global route and quantify how tightly each inferred interaction is constrained.

Although our main focus is the steady-state setting, the same construction also extends to *nonstationary* data. When all data are collected at a single time t and the time derivative $\partial_{t}\;\log\;p(x,t)$ is available, the Fokker–Planck residual acquires an additional known term, and the resulting identification problem retains the same structure. We provide this extension in the supplementary material.

In summary, our contributions are:

1. A Fokker–Planck-based formulation of system identification from non-sequential steady-state data, with two complementary routes: a local score-based method tailored to region-restricted, locally dense sampling, and a global KSD method tailored to globally sampled data with linear complexity.
2. A unified identifiability result for affine-in-parameter dynamical priors, in which both routes reduce to a linear system Aθ=b, together with a parameterwise sensitivity analysis based on the (regularized) Gram matrix $H_{\lambda }=A^{T}A+\lambda I$ that reveals which parameters are well constrained by the data.
3. Gradient-based extensions of both routes for general non-affine parameterizations.
4. Demonstrations on a stochastic Lorenz system and a nonlinear gene-regulatory network with higher-order interactions.

Together, these elements provide two first-principles routes for system-identification from non-sequential data, grounded in the FP equation, that link data, steady-state distributions, and stochastic dynamics.

### A. Related work

Existing approaches to learning stochastic dynamics can be broadly grouped by the data available: (i) *time series / trajectories*, (ii) *cross-sectional samples with time labels*, and (iii) *unordered steady-state samples without time labels*. Classical system identification from time series is mature, and sparse-regression approaches, such as SINDy, provide scalable priors for discovering governing equations.^3^ When cross-sectional measurements at multiple time points are available, pseudo-time links samples across time,^10^ and dynamical optimal transport (OT) learns time-indexed flows on Wasserstein space.^11,12^ Fokker–Planck/probability-flow viewpoints have also been used to recover dynamics from cross-sectional data.^8,13,14^

Our proposed DyNoSeD framework targets regime (iii): *non-sequential steady-state samples* with neither trajectories nor cross-sectional time labels. Accordingly, methods that rely on trajectories or time-labeled snapshots address a different data regime and are not directly comparable under our data assumptions, while remaining complementary when such supervision is available. Within this steady-state regime, we build on prior work in score estimation^16,17,19^ and Stein discrepancies.^20-22^ Our contribution is to connect these ingredients through a common Fokker–Planck residual, leading to two complementary estimators derived from the same structure (a local score-based route and a global Stein-based route), together with an explicit affine-in-parameter identifiability condition and a sensitivity analysis.

## II. PROBLEM SETUP

Consider a dynamical system governed by the Itô stochastic differential equation (SDE)

(1) $$dx=f_{\theta }(x)\;dt+G(x)\;dw_{t},$$

where $x\in R^{d}$ denotes the state, $w_{t}\in R^{d^{\prime }}$ is a standard Wiener process, G(x) is a known $d\times d^{\prime }$ matrix, and $\theta \in R^{n}$ are unknown parameters of the drift $f_{\theta }$. The diffusion coefficient is then a known positive semidefinite matrix function

(2) $$D(x)\equiv \frac{1}{2}G(x)G{\left(x\right)}^{T}.$$

For clarity of exposition in the main text, we assume a *constant* diffusion D; the state-dependent case simply adds known divergence terms in D and can be handled analogously (see the supplementary material).

The goal is to identify the dynamical parameters θ from non-sequential data. We assume that, after a transient, the SDE admits a (possibly non-equilibrium) stationary density p(x), and we observe post-transient states ${\left\{x_{i}\right\}}_{i=1}^{N}$ without time stamps. We focus on two practically common regimes: (i) samples are concentrated in several subregions of state space with possibly biased sampling rates across regions (only local information about p is available there); and (ii) samples are broadly distributed so that ${\left\{x_{i}\right\}}_{i=1}^{N}$ approximate draws from p(x), but may still be too sparse in some regions for reliable density or score estimation. The local and global routes proposed in this work are designed for these two regimes, respectively.

**FIG. 2.**
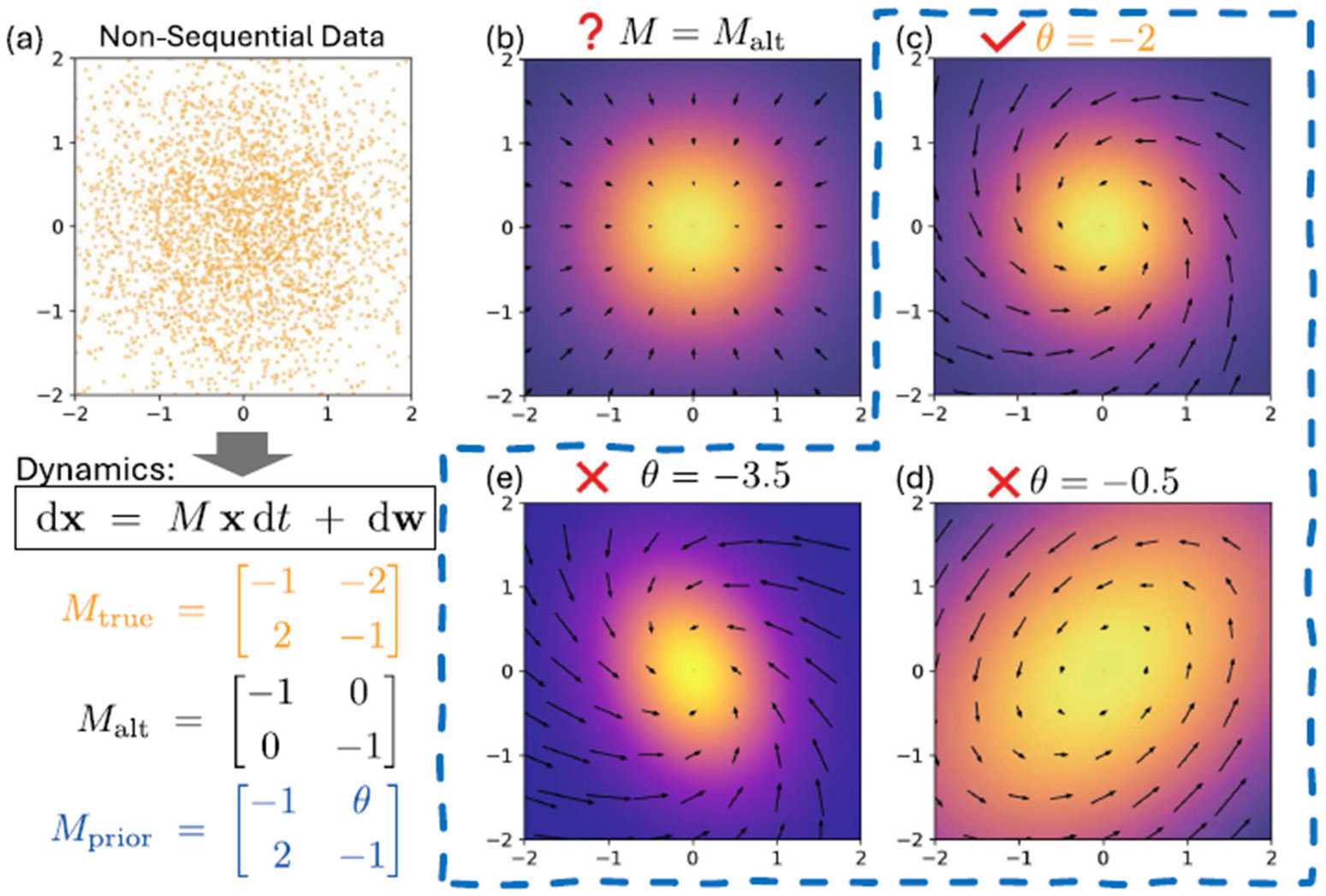
Ill-posedness from steady-state data without a constraining prior (Ornstein–Uhlenbeck example). We consider non-sequential steady-state samples from the linear SDE and try to determine the drift. (a) Unordered samples generated from the steady-state distribution of the true dynamics. (b) An alternative drift $M_{\text{alt}}$ can reproduce the same steady-state density while inducing different probability currents, illustrating non-identifiability without additional restrictions. (c) Imposing an affine prior family $M_{\text{prior}}(\theta )$ (here with a single free parameter) restores identifiability and recovers the correct parameter value (shown: θ = −2). (d) and (e) Other choices of θ produce different steady-state densities.

Without an appropriate prior structure, learning θ from such non-sequential data is generically under-determined. Even for the well-understood linear Ornstein–Uhlenbeck process (as shown by the example in Fig. 2), it is impossible to uniquely determine the drift Mx from the stationary density alone; many distinct drifts can generate the same stationary law by differing only in a divergence-free probability current. In more general nonlinear settings, identifiability becomes even more elusive due to the lack of global knowledge of the density function and the possible degeneracies in the parameterization of the drift $f_{\theta }$ under the given density function.

To make both estimation and identifiability tractable, we focus—when studying identifiability—on a practically common and analytically convenient class of priors in which the $f_{\theta }$ is an *affine-inparameter*,

(3) $$f_{\theta }(x)=U(x)\theta +v(x),$$

where $U\;:R^{d}\to R^{d\times n}$ collects n nonlinear basis vector fields (columns) encoding prior knowledge, and $v\;:R^{d}\to R^{d}$ is a known vector field. Such affine-in-parameter priors can be highly nonlinear in the state x; yet, as we show in Sec. III, they convert the identification of θ into a linear system of the form Aθ=b, with a clear necessary and sufficient condition characterizing existence and uniqueness. When no such affine-in-parameters prior is available, the methods proposed in this work can still learn θ via gradient-based optimization.

## III. SYSTEM IDENTIFICATION VIA THE FOKKER–PLANCK EQUATION

For the SDE (1) with constant diffusion D, the density p(x,t) evolves according to the Fokker–Planck equation

(4) $$\frac{\partial \;p(x,t)}{\partial t}=\nabla_{x}\cdot \left(-f_{\theta }(x)\;p(x,t)+D\;\nabla_{x}p(x,t)\right).$$

In the stationary regime of interest, $\partial_{t}p(x,t)=0$, and we define the *Fokker–Planck residual* (FPR)

(5) $$R(x;\theta )≔\nabla_{x}\cdot \left(f_{\theta }(x)\;p(x)-D\;\nabla_{x}p(x)\right),$$

which should vanish at the true parameters $\theta_{⋆}$, for all x. We now show how this residual leads to two complementary estimators: a local score-based route (Sec. III A) and a global Stein-based route (Sec. III B).

### A. Route 1: Local score-based identification

When the sampled data are restricted to, or only dense in, subregions of state space—with possibly uneven sampling across subregions—global estimation of p is not feasible. However, the *score*

(6) $$s(x)\equiv \nabla_{x}\;\log\;p(x)$$

can often be estimated *locally* from such data. Dividing (5) by p(x) and expressing derivatives in terms of the score yields a scalar *local* residual

(7) $$R_{\text{local}}(x;\theta )≔{s(x)}^{T}f_{\theta }(x)+\nabla_{x}\cdot f_{\theta }(x)-{s(x)}^{T}Ds(x)-\nabla_{x}\cdot \left(Ds(x)\right).$$

At an exact stationary solution, $R_{\text{local}}(x;\theta_{⋆})=0$ for all x. (With a *known* state-dependent diffusion matrix D(x), the corresponding local residual can be obtained from (7) by replacing D with D(x) and adding the known correction terms: $-{s(x)}^{T}\left(\nabla_{x}\cdot D(x)\right)-\nabla_{x}\cdot (\nabla_{x}\cdot D(x))$, as shown in the supplementary material.)

To infer the unknown parameters θ, we estimate the scores at *m probe locations* ${\left\{x_{i}\right\}}_{i=1}^{m}$ chosen in regions where the non-sequential data ${\left\{x_{j}\right\}}_{j=1}^{N}$ are dense, using any off-the-shelf score-estimation method, such as score matching, sliced score matching, or simple kernel-based estimators. We then minimize the local loss

(8) ℒlocal(θ)=1m∑i=1m∣Rlocal(xi;θ)∣2,

where ${\left\{x_{i}\right\}}_{i=1}^{m}$ are the probe locations. This loss can be minimized by gradient-based optimizers, such as Adam or stochastic gradient descent (SGD); when the dynamics are affine in θ, it reduces to a simple least-squares problem (Sec. III C).

### B. Route 2: Global kernel Stein discrepancy

When samples are *globally* and approximately unbiasedly drawn from the steady distribution p(x), but are too sparse to support accurate density/score estimation, we enforce the FPR condition in a global (integral) sense instead of pointwise. Specifically, we require that

(9) ∫R(x;θ)φ(x)dx=0

for all sufficiently smooth test functions φ.

Using Stein’s method, the condition (9) can be rewritten as an expectation of a differential operator acting on φ,

(10) $$E_{p}\left[{𝒜}_{\theta }^{\left(D\right)}\varphi (x)\right]=0,\;\;\forall \varphi ,$$

where the diffusion-Stein operator^23^ is defined as

(11) $${𝒜}_{\theta }^{\left(D\right)}\varphi (x)≔f_{\theta }{\left(x\right)}^{T}\nabla_{x}\varphi (x)+\text{Tr}\left(D\nabla_{x}^{2}\varphi (x)\right),$$

where $\nabla_{x}^{2}\varphi (x)$ is the Hessian of the test function. (With a *known* state-dependent diffusion matrix D(x), the corresponding operator can be obtained from (11) by simply replacing D with D(x) inside the trace term; see the supplementary material.)

Instead of checking for all test functions, we consider φ as any function within the unit ball of a universal reproducing-kernel Hilbert space (RKHS), $\varphi \in ℋ(k),\;{\left\|\varphi \right\|}_{ℋ(k)}\leq 1\;ℋ(k)$ with kernel k(x,y), and then minimize the *worst-case* violation of the Stein identity [Eq. (10)] by minimizing

(12) $$R_{\text{global}}(\theta )≔\underset{\varphi \in ℋ(k),{\left\|\varphi \right\|}_{ℋ(k)}\leq 1}{\text{sup}}{\left(E_{x∼p}[{𝒜}_{\theta }^{\left(D\right)}\varphi (x)]\right)}^{2}.$$

Based on the reproducing property, φ(x) can be rewritten as ${\langle \varphi (\cdot ),k(x,\cdot )\rangle }_{ℋ}$. Thus, by applying the sample mean and the differential operator to φ(⋅), we obtain

(13) $$E_{x∼p}\left[{𝒜}_{\theta }^{\left(D\right)}\varphi (x)\right]={\langle \varphi (\cdot ),E_{x∼p}\left[{𝒜}_{\theta }^{\left(D\right)}k(x,\cdot )\right]\rangle }_{ℋ}.$$

By using the Schwarz inequality, the fact that ${\langle \varphi (\cdot ),\varphi (\cdot )\rangle }_{ℋ}\leq 1$, and the identity ${\langle k(x,\cdot ),k(y,\cdot )\rangle }_{ℋ}=k(x,y)$, we obtain

(14) $$R_{\text{global}}(\theta )≔{\left\|E_{x∼p}\left[{𝒜}_{\theta }^{\left(D\right)}k(x,\cdot )\right]\right\|}_{ℋ}^{2}=\frac{1}{N^{2}}\sum_{i,j=1}^{N}{𝒜}_{\theta ,x_{i}}^{\left(D\right)}{𝒜}_{\theta ,x_{j}}^{\left(D\right)}k(x_{i},x_{j}).$$

The computational complexity for obtaining $R_{\text{global}}(\theta )$ directly from (14) is $O(N^{2})$; i.e., it is quadratic in the number of observations. In practice, we use a linear complexity method to minimize the KSD by choosing a shift-invariant Gaussian RBF kernel and approximating it with m random Fourier features via Bochner’s theorem. Drawing frequencies ${\left\{\omega_{r}\right\}}_{r=1}^{m}∼𝒩(0,\ell^{-2}I_{d})$ and phases ${\left\{c_{r}\right\}}_{r=1}^{m}∼\text{Unif}[0,2\pi ]$, we define

(15) $$z_{r}(x)≔\sqrt{\frac{2}{m}}\;\cos\left(\omega_{r}^{T}x+c_{r}\right),\;z(x)≔{\left(z_{1}(x),\ldots ,z_{m}(x)\right)}^{T},$$

so that $k(x,y)\approx z{\left(x\right)}^{T}z(y)$ for large m.

Applying the Stein operator (11) to each feature defines the *Stein feature vector*

(16) $$g(x;\theta )≔({𝒜}_{\theta }^{\left(D\right)}z)(x)≔\left[\begin{array}{c} {𝒜}_{\theta }^{\left(D\right)}z_{1}(x)\\ \vdots \\ {𝒜}_{\theta }^{\left(D\right)}z_{m}(x) \end{array}\right]\in R^{m},$$

with components

(17) $${\left[g(x;\theta )\right]}_{r}=-\sqrt{\frac{2}{m}}\left[\sin\left(\omega_{r}^{T}x+c_{r}\right)\omega_{r}^{T}f_{\theta }(x)+\cos\left(\omega_{r}^{T}x+c_{r}\right)\omega_{r}^{T}D\;\omega_{r}\right].$$

The diffusion-Stein identity implies that, at the true parameters, $E_{x∼p}[g(x;\theta_{⋆})]=0$. Given globally sampled (non-sequential) data ${\left\{x_{i}\right\}}_{i=1}^{N}∼p$, we, therefore, define the *global* KSD loss as the squared norm of the empirical mean Stein feature,

(18) ℒglobal(θ)≔‖1N∑i=1Ng(xi;θ)‖22.

This objective has linear complexity O(Nm) in the number of samples N and features m and can be minimized over θ using standard gradient-based optimizers. In the affine-in-parameters case, the mean Stein feature is linear in θ, and in the infinite-data limit, the root condition $E_{x∼p}[g(x;\theta )]=0$ again reduces to a linear system (Sec. III C).

### C. Identification condition in the affine-in-parameter case

When the drift is affine in the unknown parameters θ as in Eq. (3), both routes induce linear systems of the form Aθ=b.

For the *local* route, substituting $f_{\theta }$ from (3) into the local residual (7) and using an estimated score $\hat{s}(x_{i})$ at each probe $x_{i}$ yields a scalar equation

(19) $$a{\left(x_{i}\right)}^{T}\theta =b(x_{i}),$$

where

(20) $$a(x_{i})≔U{\left(x_{i}\right)}^{T}\hat{s}(x_{i})+\nabla_{x}\cdot U(x_{i}),$$

(21) $$b(x_{i})≔{\hat{s}(x_{i})}^{T}D\hat{s}(x_{i})+\nabla_{x}\cdot \left(D\hat{s}(x_{i})\right)-{\hat{s}(x_{i})}^{T}v(x_{i})-\nabla_{x}\cdot v(x_{i}).$$

Stacking m probes gives the linear system

(22) $$A_{\text{local}}\theta =b_{\text{local}},$$

with rows ${a(x_{i})}^{T}$ and entries $b(x_{i})$.

For the *global* route, substituting Eq. (3) into Eq. (18) yields

(23) $$E_{x∼p}\left[\sin\left(\omega_{r}^{T}x+c_{r}\right)\omega_{r}^{T}U(x)\right]\theta \;\;=E_{x∼p}\left[\sin\left(\omega_{r}^{T}x+c_{r}\right)\omega_{r}^{T}v(x)+\cos\left(\omega_{r}^{T}x+c_{r}\right)\omega_{r}^{T}D\omega_{r}\right]$$

for r=1,…,m. Stacking m features gives the linear system

(24) $$A_{\text{global}}\theta =b_{\text{global}},$$

with each row of $A_{\text{global}}$ and each entry $b_{\text{global}}$ being defined in Eq. (23).

In both routes, we, thus, obtain a linear system

(25) Aθ=b,

where A and b denote either the local or global matrices/vectors above. The existence and uniqueness of θ are characterized by a simple rank condition:

**Theorem 1 (Identification in the affine-in-parameter case):** *Let* $A\in R^{M\times n}$ *and* $b\in R^{M}$ *be the matrix and vector obtained from either the local score route or the global Stein route, under exact scores (local) or infinite data (global). Then, there exists a parameter vector* θ *whose dynamics satisfy the corresponding Fokker–Planck constraints if and only if* b∈range(A); *this solution is unique if and only if* rank(A)=n.

The proof is immediate from linear algebra: existence of a solution is equivalent to b belonging to the column space of A, range(A), and uniqueness requires a trivial null space, ker(A)={0}, i.e., a full column rank.

For the affine-in-parameter case, we could practically infer θ by solving the regularized least-squares problem using

(26) $${\hat{\theta }}_{\lambda }={\left(A^{T}A+\lambda I\right)}^{T}A^{T}b.$$

To handle the over-parameterization, we use the (regularized) Gram matrix

(27) $$H_{\lambda }≔A^{T}A+\lambda I,$$

which encodes how well each of the inferred parameters in $\hat{\theta }$ is constrained.

## IV. DEMONSTRATIONS

We illustrate DyNoSeD on two stochastic systems with very different structures.

### A. Stochastic Lorenz system

We first consider the classical Lorenz SDE $dx_{t}=f(x_{t};\theta )\;dt+\sqrt{2D}\;dw_{t}$ with parameters θ=(σ,ρ,β) and additive isotropic noise. We simulate long trajectories at the true parameters and thin them to obtain non-sequential steady-state samples.

**FIG. 3.**
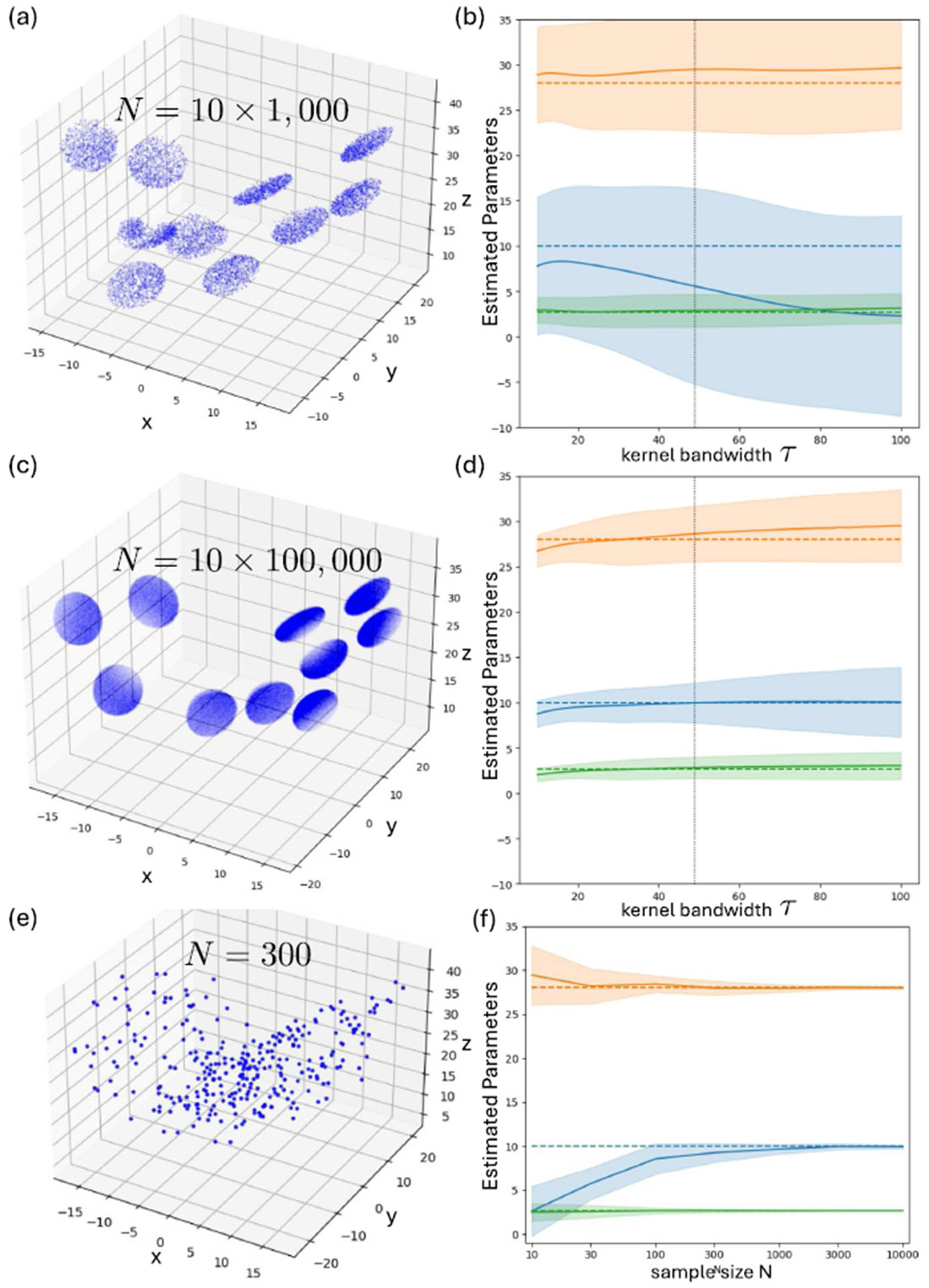
Lorenz SDE: local vs global identification from non-sequential steady-state samples. (a), (c), and (e) Examples of unordered steady-state samples on the Lorenz attractor under three sampling regimes: region-restricted data consisting of *m* = 10 locally dense spheres with 10^3^ points per sphere (*N* = 10 × 10^3^) in (a), the same construction with 10^5^ points per sphere (*N* = 10 × 10^5^) in (c), and a globally sampled but sparse cloud (*N* = 300) in (e). (b) and (d) *Local score-based route:* recovered parameters (σ, ρ, β) as a function of Gaussian kernel bandwidth 𝒯 using the region-restricted datasets in (a) and (c); solid curves denote the mean over 50 trials and shaded bands denote one standard deviation, with dashed lines indicating ground truth (vertical dotted line: reference bandwidth matched with the negative Lyapunov exponent of the Lorenz system). (f) *Global KSD route:* recovered parameters as a function of sample size *N* from globally sampled steady-state data, showing accurate recovery with only a few hundred points.

For the *local* route, we estimate scores at the centers of *m* = 10 spheres using only the samples within each sphere, via a Gaussian kernel with bandwidth 𝒯. Specifically, we construct the region-restricted data and choose probe locations as follows, repeating the procedure for 50 random trials. In each trial, we repeatedly draw ten distinct candidate points uniformly from the full non-sequential dataset and accept them only if (i) all pairwise distances among the candidates exceed 2r with r = 4 (so the spheres do not overlap), and (ii) each sphere $B(c_{j},r)$ centered at a candidate $c_{j}$ contains at least $N_{\text{min}}$ samples from the full dataset [$N_{\text{min}}$ = 1000 for Figs. 3(a) and 3(b) and $N_{\text{min}}$ = 100,000 for Figs. 3(c) and 3(d)]. Once an accepted set ${\left\{c_{j}\right\}}_{j=1}^{10}$ is obtained, we fix these centers as probe locations and form the trial-specific region-restricted dataset by uniformly subsampling exactly $N_{\text{loc}}\in \{10^{3},10^{5}\}$ points from within each sphere. Thus, each trial uses an intentionally non-uniform, region-restricted subset of steady-state samples. Comparing Figs. 3(b) and 3(d), when each sphere contains more points, the local route recovers all three parameters accurately over a broad range of 𝒯. We also note that the score quality depends on the kernel bandwidth; for reference, we mark the near-optimal 𝒯 ≈ 49 in Figs. 3(b) and 3(d) with vertical dashed lines.^24^ When the persphere sample size is small [Figs. 3(a) and 3(b)], the estimated scores degrade, and the inferred parameters become biased and sensitive to 𝒯.

For the *global* route, we use all globally sampled data as a single cloud and minimize the linear-time KSD loss without explicit score estimation. Even with only N = 300 globally sampled points, the KSD route gives nearly unbiased estimates for all three parameters, and the variance shrinks rapidly with N [Figs. 3(e) and 3(f)]. This highlights the complementary regimes of the two routes: local scores are powerful when data are dense in targeted regions, while the global KSD route is robust under sparse, broadly distributed sampling.

### B. Nonlinear gene-regulatory network

Next, we study a seven-dimensional SDE derived from a published B-cell differentiation model.^18^ Three genes (p, b, r) are regulated by themselves and by two housekeeping pathways (BCR and CD40). We encode regulation through third-order interactions of the transformed activities $\pi =1∕(1+p^{2})$, $\beta =1∕(1+b^{2})$, $\rho =1∕(1+r^{2})$, i.e., π, β, ρ, πβ, πρ, βρ, and πβρ, yielding a 3 × 7 interaction matrix. Four additional variables describe autonomous BCR/CD40 oscillators, leading to a coupled seven-dimensional SDE. We simulate long and stochastic trajectories, thin them to obtain unordered steady-state samples, and apply the *global* KSD route in its analytic affine form to recover the interaction matrix.

**FIG. 4.**
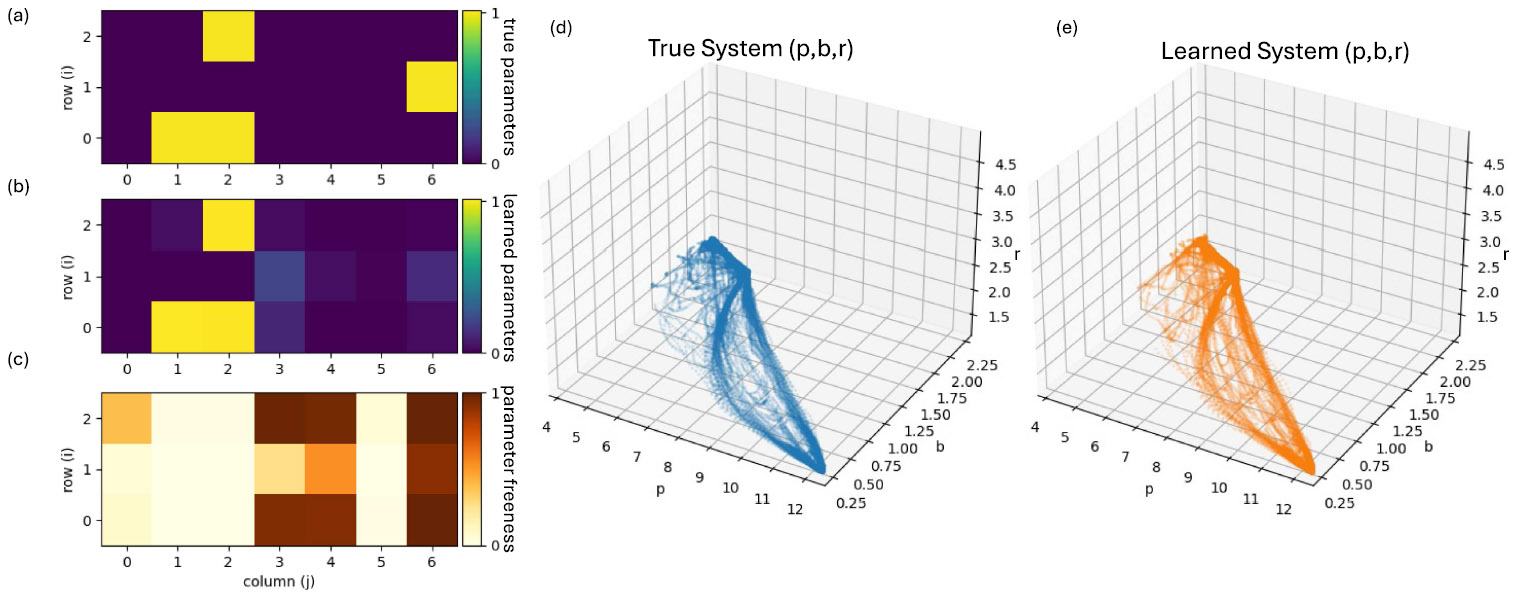
Nonlinear gene-regulatory network: parameter recovery and freeness from unordered steady-state samples. (a) Ground-truth 3 × 7 interaction matrix [rows correspond to (p, b, r) and columns to the seven regulatory inputs]. (b) Interaction matrix inferred from non-sequential steady-state data using the global KSD route. (c) Normalized parameter *freeness* (larger values indicate directions that are weakly constrained by the data), computed from the diagonal of the regularized inverse Gram matrix $H_{\lambda }^{-1}$. The largest mismatch between (a) and (b) aligns with a high-freeness entry in (c). (d) and (e) Steady-state clouds in the (p, b, r) subspace generated by simulating the true system (d) and the learned system (e), showing visually similar stationary behavior despite localized parameter uncertainty.

Figures 4(a) and 4(b) show the true and the inferred 3 × 7 parameters. With λ = 10^−6^, most nonzero entries are recovered with small errors, but one interaction [last element in the second row in Fig. 4(b)] is clearly misestimated. To understand this, we examine the Hessian matrix [Eq. (27)] and compute parameter-wise “freeness” from the diagonal of $H_{\lambda }^{-1}$; small freeness indicates that a parameter is tightly constrained by the data, while large freeness indicates an effectively free parameter not constrained by the data. The resulting heatmap (bottom-right panel) reveals that the misestimated interaction lies in one of the least constrained directions, consistent with the linear sensitivity analysis.

Crucially, the true and the learned dynamical systems generate visually indistinguishable steady-state clouds in the (p, b, r) subspace [Figs. 4(d) and 4(e)], even though individual poorly constrained parameters differ. This illustrates how DyNoSeD, together with the Gram-based sensitivity analysis, can separate parameters that are reliably identified from those that are effectively free under over-parameterization.

## V. CONCLUSION

We introduced DyNoSeD, a Fokker–Planck-based framework for identifying stochastic dynamics from non-sequential data. By deriving both a local score-based route and a global Stein-based route from the same FP residual, we can handle region-restricted dense sampling and globally sparse sampling within a unified formulation. In the affine-in-parameter case, both routes reduce to a linear system Aθ=b, yielding a simple rank-based identifiability condition and a Gram-matrix sensitivity analysis that reveals which parameters are well constrained and which are effectively free.

Our demonstrations on the Lorenz system and a nonlinear gene-regulatory network show that DyNoSeD can recover both low-dimensional and over-parameterized dynamics from unordered steady-state samples, and that the sensitivity analysis provides interpretable parameterwise reliability. We expect these ideas to be useful in applications where only snapshot measurements are available, and where understanding which aspects of a mechanistic model are truly constrained by such data is as important as fitting the model itself.

## Supplementary Material

Supplementary Material

See the supplementary material for additional proofs and implementation details. In all experiments in this paper, we estimate s using a simple Gaussian radial kernel estimator with a bandwidth (“temperature”) parameter 𝒯; the examples show that the recovered parameters are robust over a broad range of 𝒯 when the data are locally dense.

## Data Availability

The data that support the findings of this study are available from the corresponding author upon reasonable request.
