# Supplementary material for: Identifying stochastic dynamics from non-sequential data (DyNoSeD)
Source: Chaos. Author manuscript; Available in PMC 2026 May 21. (PMC13193673; doi:10.1063/5.0314136)
Supplement: Supplementary Material [file NIHMS2171656-supplement-Supplementary_Material.pdf]

SUPPLEMENTARY MATERIAL

S1. DERIVING LOCAL ROUTE FOR THE MOST GENERAL CASE

S1.1. Local route for nonstationary data

In this section we extend the local route to the more general setting of *nonstationary* data. We consider time–varying SDEs

$$d\mathbf{x}_t = \mathbf{f}_{\boldsymbol{\theta}}(\mathbf{x}_t, t)\, dt + \mathbf{G}(\mathbf{x}_t, t)\, d\mathbf{w}_t, \tag{S1}$$

with state– and time–dependent diffusion

$$\boldsymbol{D}(\mathbf{x}, t) := \tfrac{1}{2}\, \mathbf{G}(\mathbf{x}, t)\mathbf{G}(\mathbf{x}, t)^{\top}. \tag{S2}$$

Denote the i–th component of $\mathbf{f}_{\boldsymbol{\theta}}$ by f_i. If $p(\mathbf{x}, t)$ is the time–dependent density of $\mathbf{x}_t$, then p obeys the Fokker–Planck equation

$$\begin{aligned} \frac{\partial p(\mathbf{x}, t)}{\partial t} &= -\sum_{i=1}^{d} \frac{\partial}{\partial x_i}\big[f_i(\mathbf{x}, t)\, p(\mathbf{x}, t)\big] + \sum_{i=1}^{d}\sum_{j=1}^{d} \frac{\partial^2}{\partial x_i \partial x_j}\big[\boldsymbol{D}_{ij}(\mathbf{x}, t)\, p(\mathbf{x}, t)\big] && \text{(S3)}\\ &= \sum_{i=1}^{d} \frac{\partial}{\partial x_i}\Big(- f_i(\mathbf{x}, t)\, p(\mathbf{x}, t) + \sum_{j=1}^{d} \frac{\partial}{\partial x_j}[\boldsymbol{D}_{ij}(\mathbf{x}, t)\, p(\mathbf{x}, t)]\Big). && \text{(S4)} \end{aligned}$$

Expanding the inner term and introducing the score $\mathbf{s}(\mathbf{x}, t) := \nabla_{\mathbf{x}} \log p(\mathbf{x}, t)$, we obtain

$$\begin{aligned} \sum_{j=1}^{d} \frac{\partial}{\partial x_j}[\boldsymbol{D}_{ij}(\mathbf{x}, t)\, p(\mathbf{x}, t)] &= \sum_{j=1}^{d} \Big(\frac{\partial \boldsymbol{D}_{ij}}{\partial x_j}(\mathbf{x}, t)\, p(\mathbf{x}, t) + \boldsymbol{D}_{ij}(\mathbf{x}, t)\, \frac{\partial p}{\partial x_j}(\mathbf{x}, t)\Big) && \text{(S5)}\\ &= p(\mathbf{x}, t)\, \big(\nabla_{\mathbf{x}} \cdot \boldsymbol{D}(\mathbf{x}, t)\big)_i + p(\mathbf{x}, t)\, \big(\boldsymbol{D}(\mathbf{x}, t)\mathbf{s}(\mathbf{x}, t)\big)_i, && \text{(S6)} \end{aligned}$$

where we define the matrix divergence

$$\big(\nabla_{\mathbf{x}} \cdot \boldsymbol{D}(\mathbf{x}, t)\big)_i := \sum_{j=1}^{d} \frac{\partial \boldsymbol{D}_{ij}}{\partial x_j}(\mathbf{x}, t).$$

In vector notation, Eq. (S4) becomes

$$\frac{\partial p(\mathbf{x}, t)}{\partial t} = -\,\nabla_{\mathbf{x}} \cdot \Big(\mathbf{f}_{\boldsymbol{\theta}}(\mathbf{x}, t)\, p(\mathbf{x}, t) - p(\mathbf{x}, t)\, \nabla_{\mathbf{x}} \cdot \boldsymbol{D}(\mathbf{x}, t) - p(\mathbf{x}, t)\, \boldsymbol{D}(\mathbf{x}, t)\mathbf{s}(\mathbf{x}, t)\Big). \tag{S7}$$

Introduce

$$\mathbf{F}^{(\boldsymbol{D})}_{\boldsymbol{\theta}}(\mathbf{x}, t) := \mathbf{f}_{\boldsymbol{\theta}}(\mathbf{x}, t) - \nabla_{\mathbf{x}} \cdot \boldsymbol{D}(\mathbf{x}, t) - \boldsymbol{D}(\mathbf{x}, t)\mathbf{s}(\mathbf{x}, t), \tag{S8}$$

so that Eq. (S7) reads

$$\frac{\partial p(\mathbf{x}, t)}{\partial t} = -\,\nabla_{\mathbf{x}} \cdot \big(p(\mathbf{x}, t)\, \mathbf{F}^{(\boldsymbol{D})}_{\boldsymbol{\theta}}(\mathbf{x}, t)\big). \tag{S9}$$

Assuming $p(\mathbf{x}, t) > 0$ on the region of interest, divide both sides by p:

$$\frac{\partial}{\partial t} \log p(\mathbf{x}, t) = -\,\frac{1}{p(\mathbf{x}, t)}\, \nabla_{\mathbf{x}} \cdot \big(p(\mathbf{x}, t)\, \mathbf{F}^{(\boldsymbol{D})}_{\boldsymbol{\theta}}(\mathbf{x}, t)\big). \tag{S10}$$

Using $\nabla_{\mathbf{x}} p = p\,\mathbf{s}$ and the product rule,

$$\nabla_{\mathbf{x}} \cdot \big(p\mathbf{F}^{(\boldsymbol{D})}_{\boldsymbol{\theta}}\big) = (\mathbf{F}^{(\boldsymbol{D})}_{\boldsymbol{\theta}})^{\top} \nabla_{\mathbf{x}} p + p \nabla_{\mathbf{x}} \cdot \mathbf{F}^{(\boldsymbol{D})}_{\boldsymbol{\theta}} = p\big(\mathbf{s}^{\top} \mathbf{F}^{(\boldsymbol{D})}_{\boldsymbol{\theta}} + \nabla_{\mathbf{x}} \cdot \mathbf{F}^{(\boldsymbol{D})}_{\boldsymbol{\theta}}\big),$$

we arrive at

$$\frac{\partial}{\partial t}\log p(\mathbf{x},t)+\mathbf{s}(\mathbf{x},t)^\top\mathbf{F}_{\boldsymbol{\theta}}^{(\boldsymbol{D})}(\mathbf{x},t)+\nabla_{\mathbf{x}}\cdot\mathbf{F}_{\boldsymbol{\theta}}^{(\boldsymbol{D})}(\mathbf{x},t)=0. \tag{S11}$$

Equivalently,

$$\frac{\partial}{\partial t}\log p(\mathbf{x},t)+\mathbf{s}(\mathbf{x},t)^\top\big(\mathbf{f}_{\boldsymbol{\theta}}-\nabla_{\mathbf{x}}\cdot\boldsymbol{D}-\boldsymbol{D}\mathbf{s}\big)+\nabla_{\mathbf{x}}\cdot\big(\mathbf{f}_{\boldsymbol{\theta}}-\nabla_{\mathbf{x}}\cdot\boldsymbol{D}-\boldsymbol{D}\mathbf{s}\big)=0. \tag{S12}$$

We define the *nonstationary Fokker–Planck residual* at $(\mathbf{x},t)$ by

$$\begin{aligned}R(\mathbf{x},t;\boldsymbol{\theta}):=&\partial_t\log p(\mathbf{x},t)+\\&\mathbf{s}(\mathbf{x},t)^\top\big(\mathbf{f}_{\boldsymbol{\theta}}(\mathbf{x},t)-\nabla_{\mathbf{x}}\cdot\boldsymbol{D}(\mathbf{x},t)-\boldsymbol{D}(\mathbf{x},t)\mathbf{s}(\mathbf{x},t)\big)+\\&\nabla_{\mathbf{x}}\cdot\big(\mathbf{f}_{\boldsymbol{\theta}}(\mathbf{x},t)-\nabla_{\mathbf{x}}\cdot\boldsymbol{D}(\mathbf{x},t)-\boldsymbol{D}(\mathbf{x},t)\mathbf{s}(\mathbf{x},t)\big),\end{aligned} \tag{S13}$$

so that $R(\mathbf{x},t;\boldsymbol{\theta}^*)=0$ for the true parameters $\boldsymbol{\theta}^*$.

Suppose we have a collection of measurements at (possibly one or multiple) time points,

$$\Omega=\{(\mathbf{x}_i,t_i)\}_{i=1}^N,$$

together with local estimates of the score $\mathbf{s}(\mathbf{x}_i,t_i)$ and the time derivative $\partial_t\log p(\mathbf{x}_i,t_i)$ (e.g., from a parametric or neural density model). The local nonstationary route then fits $\boldsymbol{\theta}$ by minimizing the empirical FP residual, e.g. via

$$\mathcal{L}_{\text{local}}(\boldsymbol{\theta};\Omega):=\frac{1}{N}\sum_{i=1}^N\Big(R(\mathbf{x}_i,t_i;\boldsymbol{\theta})\Big)^2, \tag{S14}$$

which reduces to the steady-state local loss when $\partial_t\log p\equiv 0$.

S2. GENERAL DIFFUSION–STEIN OPERATOR AND LINEAR–COMPLEXITY KSD

S2.1. Diffusion–Stein operator for state–dependent, non–stationary SDEs

Consider the time-dependent Itô SDE with state– and time–dependent diffusion matrix as specified in Eq. S1. Let $p(\mathbf{x},t)$ denote the density of $\mathbf{x}_t$. The associated Fokker-Planck equation is

$$\frac{\partial p(\mathbf{x},t)}{\partial t}+\nabla_{\mathbf{x}}\cdot\Big(\mathbf{f}_{\boldsymbol{\theta}}(\mathbf{x},t)\,p(\mathbf{x},t)-\nabla_{\mathbf{x}}\cdot\big(\boldsymbol{D}(\mathbf{x},t)p(\mathbf{x},t)\big)\Big)=0. \tag{S15}$$

Let $\varphi:\mathbb{R}^d\to\mathbb{R}$ be a smooth test function with sufficient decay so that boundary terms vanish under integration by parts. Multiplying (S15) by $\varphi(\mathbf{x})$ and integrating over $\mathbf{x}$ gives

$$\int\Big[\frac{p(\mathbf{x},t)}{p(\mathbf{x},t)}\frac{\partial p(\mathbf{x},t)}{\partial t}+\nabla_{\mathbf{x}}\cdot\Big(\mathbf{f}_{\boldsymbol{\theta}}(\mathbf{x},t)\,p(\mathbf{x},t)-\nabla_{\mathbf{x}}\cdot\big(\boldsymbol{D}(\mathbf{x},t)p(\mathbf{x},t)\big)\Big]\varphi(\mathbf{x})\mathrm{d}\mathbf{x}=0 \tag{S16}$$

By applying integration-by-parts (i.e., the Divergence Theorem) multiple times, we could obtain

$$-\int p(\mathbf{x},t)\,\varphi(\mathbf{x})\,\partial_t\log p(\mathbf{x},t)\,\mathrm{d}\mathbf{x}\;+\int p(\mathbf{x},t)\,\Big[\mathbf{f}_{\boldsymbol{\theta}}(\mathbf{x},t)\cdot\nabla_{\mathbf{x}}\varphi(\mathbf{x})+\mathrm{Tr}\big(\boldsymbol{D}(\mathbf{x},t)\,\nabla_{\mathbf{x}}^2\varphi(\mathbf{x})\big)\Big]\mathrm{d}\mathbf{x}=0, \tag{S17}$$

which leads to

$$\mathbb{E}_{\mathbf{x}\sim p(\mathbf{x},t)}\big[-\big(\partial_t\log p(\mathbf{x},t)\big)\varphi(\mathbf{x})+\mathcal{A}_{\boldsymbol{\theta},t}^{(\boldsymbol{D})}\varphi(\mathbf{x})\big]=0, \tag{S18}$$

where we have defined the diffusion–Stein operator

$$\mathcal{A}_{\boldsymbol{\theta},t}^{(\boldsymbol{D})}\varphi(\mathbf{x}):=\mathbf{f}_{\boldsymbol{\theta}}(\mathbf{x},t)\cdot\nabla_{\mathbf{x}}\varphi(\mathbf{x})+\mathrm{Tr}\big(\boldsymbol{D}(\mathbf{x},t)\,\nabla_{\mathbf{x}}^2\varphi(\mathbf{x})\big). \tag{S19}$$

For constant diffusion, $\boldsymbol{D}(\mathbf{x},t)\equiv D$, the $\mathcal{A}_{\boldsymbol{\theta},t}^{(\boldsymbol{D})}$ reduces to the operator $\mathcal{A}_{\boldsymbol{\theta}}^{(D)}$ used in the main text (Eq. 11).

Two special cases are of particular interest:

- **Stationary regime.** If $p(\mathbf{x},t)$ has reached a stationary density $p^*(\mathbf{x})$, then $\partial_t p(\mathbf{x},t) = 0$ and $\partial_t \log p(\mathbf{x},t) = 0$, so (S18) reduces to the standard diffusion–Stein identity

$$\mathbb{E}_{\mathbf{x}\sim p^*}\big[\mathcal{A}_{\boldsymbol{\theta}}^{(\boldsymbol{D})}\varphi(\mathbf{x})\big] = 0, \qquad \forall\,\varphi, \tag{S20}$$

with $\mathcal{A}_{\boldsymbol{\theta}}^{(\boldsymbol{D})} := \mathcal{A}_{\boldsymbol{\theta},t}^{(\boldsymbol{D})}$ evaluated at stationarity.

- **Non–stationary snapshot.** Fix a time t_0 and suppose we have access to $\partial_t \log p(\mathbf{x},t_0)$ (e.g. from a density model, which does not require sequential data as it only needs the changing rate of the log likelihood at each provided data point). Define the augmented Stein operator

$$\mathcal{B}_{\boldsymbol{\theta},t_0}\varphi(\mathbf{x}) := -\varphi(\mathbf{x})\,\partial_t \log p(\mathbf{x},t_0) + \mathcal{A}_{\boldsymbol{\theta},t_0}^{(\boldsymbol{D})}\varphi(\mathbf{x}). \tag{S21}$$

Then (S18) can be written compactly as

$$\mathbb{E}_{\mathbf{x}\sim p(\mathbf{x},t_0)}\big[\mathcal{B}_{\boldsymbol{\theta},t_0}\varphi(\mathbf{x})\big] = 0, \qquad \forall\,\varphi, \tag{S22}$$

which generalizes the stationary Stein identity to a non–stationary snapshot at t_0.

In the main text we focus on the stationary case with constant diffusion, so $\mathcal{B}_{\boldsymbol{\theta},t_0}$ reduces to $\mathcal{A}_{\boldsymbol{\theta}}^{(D)}$ and (S22) becomes Eq. 10 in the main text.

S2.2. Kernel Stein discrepancy

Let $k(\mathbf{x},\mathbf{y})$ be a positive–definite kernel with RKHS $\mathcal{H}(k)$ and reproducing property

$$\varphi(\mathbf{x}) \;=\; \langle \varphi(\cdot),\, k(\mathbf{x},\cdot)\rangle_{\mathcal{H}(k)} \qquad \text{for all } \varphi\in\mathcal{H}(k).$$

We then let $\mathcal{B}_{\boldsymbol{\theta},t_0}$, the (possibly nonstationary) Stein operator for the most general case given above, be applied on to $\varphi(\mathbf{x})$, the reproducing property yields,

$$\mathcal{B}_{\boldsymbol{\theta},t_0}\varphi(\mathbf{x}) \;=\; \langle \varphi(\cdot),\, \mathcal{B}_{\boldsymbol{\theta},t_0}k(\mathbf{x},\cdot)\rangle_{\mathcal{H}(k)} \qquad \text{for all } \varphi\in\mathcal{H}(k).$$

Now, we take the sample mean and obtain

$$\mathbb{E}_{\mathbf{x}\sim p(\mathbf{x},t)}[\mathcal{B}_{\boldsymbol{\theta},t_0}\varphi(\mathbf{x})] \;=\; \langle \varphi(\cdot),\, \mathbb{E}_{\mathbf{x}\sim p(\mathbf{x},t)}[\mathcal{B}_{\boldsymbol{\theta},t_0}k(\mathbf{x},\cdot)]\rangle_{\mathcal{H}(k)} \qquad \text{for all } \varphi\in\mathcal{H}(k).$$

Now, let's only consider $\varphi\in\mathcal{H}_k$ that is within the surface of the unit ball, i.e., $\|\varphi\|_{\mathcal{H}}\le 1$. Then, by using the Schwarz inequality, we obtain

$$\Big(\langle \varphi(\cdot),\, \mathbb{E}_{\mathbf{x}\sim p(\mathbf{x},t)}[\mathcal{B}_{\boldsymbol{\theta},t_0}k(\mathbf{x},\cdot)]\rangle_{\mathcal{H}(k)}\Big)^2 \le \|\varphi(\cdot)\|_{\mathcal{H}}^2\;\|\mathbb{E}_{\mathbf{x}\sim p(\mathbf{x},t)}[\mathcal{B}_{\boldsymbol{\theta},t_0}k(\mathbf{x},\cdot)]\|_{\mathcal{H}}^2. \tag{S23}$$

Thus, we are guaranteed that worst squared kernel Stein discrepancy in Eq. S22 is bounded by the inequality,

$$\big(\mathbb{E}_{\mathbf{x}\sim p(\mathbf{x},t_0)}\big[\mathcal{B}_{\boldsymbol{\theta},t_0}\varphi(\mathbf{x})\big]\big)^2 \;\le\; \langle \mathbb{E}_{\mathbf{x}\sim p(\mathbf{x},t_0)}\big[\mathcal{B}_{\boldsymbol{\theta},t_0;\mathbf{x}}k(\mathbf{x},\cdot)\big], \mathbb{E}_{\mathbf{y}\sim p(\mathbf{y},t_0)}\big[B_{\boldsymbol{\theta},t_0;\mathbf{y}}k(\mathbf{y},\cdot)\big]\rangle_{\mathcal{H}(k)}. \tag{S24}$$

By taking the sample mean out, we obtain

$$\langle \mathbb{E}_{\mathbf{x}\sim p(\mathbf{x},t_0)}\big[B_{\boldsymbol{\theta},t_0;\mathbf{x}}k(\mathbf{x},\cdot)\big], \mathbb{E}_{\mathbf{y}\sim p(\mathbf{y},t_0)}\big[B_{\boldsymbol{\theta},t_0;\mathbf{y}}k(\mathbf{y},\cdot)\big]\rangle_{\mathcal{H}(k)} = \mathbb{E}_{\mathbf{x},\mathbf{y}}\Big[\big\langle \mathcal{B}_{\boldsymbol{\theta},t_0,\mathbf{x}}k(\mathbf{x},\cdot),\, \mathcal{B}_{\boldsymbol{\theta},t_0,\mathbf{y}}k(\mathbf{y},\cdot)\big\rangle_{\mathcal{H}(k)}\Big], \tag{S25}$$

where $\mathbf{x},\mathbf{y}\sim p(\cdot,t_0)$ independently. Here, we guarantee that for any fixed $\mathbf{y}$, the function $\mathcal{B}_{\boldsymbol{\theta},t_0,\mathbf{y}}k(\mathbf{y},\cdot)$ remains in $\mathcal{H}(k)$ by choosing kernel that is universal. Then, by applying the two operators in Eq. S25 (one on $\mathbf{x}$ and the other on $\mathbf{y}$) onto the reproducing property,

$$k(x,y) = \langle k(\mathbf{x},\cdot), k(\mathbf{y},\cdot)\rangle_{\mathcal{H}}$$

Eqs. S24-S25 yields

$$\mathrm{KSD}^2(\boldsymbol{\theta}) = \mathbb{E}_{\mathbf{x},\mathbf{y}\sim p(\cdot,t_0)}\big[k_{\boldsymbol{\theta}}(\mathbf{x},\mathbf{y})\big], \tag{S26}$$

with the *Stein kernel*

$$k_{\boldsymbol{\theta}}(\mathbf{x},\mathbf{y}) := \mathcal{B}_{\boldsymbol{\theta},t_0,\mathbf{x}}\mathcal{B}_{\boldsymbol{\theta},t_0,\mathbf{y}}k(\mathbf{x},\mathbf{y}). \tag{S27}$$

Given i.i.d. samples $\{\mathbf{x}_i\}_{i=1}^N \sim p(\cdot,t_0)$, the standard estimator of (S26) is

$$\widehat{\mathrm{KSD}}^2(\boldsymbol{\theta}) := \frac{1}{N^2}\sum_{i,j=1}^{N} k_{\boldsymbol{\theta}}(\mathbf{x}_i,\mathbf{x}_j), \tag{S28}$$

which reduces to Eq. 14 in the main text when $\partial_t \log p(\mathbf{x},t_0) \equiv 0$ and $\boldsymbol{D}(\mathbf{x},t_0) \equiv D$ is constant. The direct computation of (S28) requires $O(N^2)$ time; in the next subsection we show how to obtain a linear complexity approximation using random Fourier features.

S2.3. Random Fourier features (RFFs) and linear–complexity KSD

To obtain a linear–time approximation, we specialize to a shift–invariant kernel $k(\mathbf{x},\mathbf{y}) = k(\mathbf{x}-\mathbf{y})$ with spectral density $p(\boldsymbol{\omega})$ and use random Fourier features. Here, we consider the Gaussian RBF kernel

$$k(\mathbf{x},\mathbf{y}) = \exp\Big(-\frac{\|\mathbf{x}-\mathbf{y}\|_2^2}{2\ell^2}\Big). \tag{S29}$$

Bochner's theorem states that any continuous, positive–definite, shift–invariant kernel admits the representation

$$k(\mathbf{x}-\mathbf{y}) = \int_{\mathbb{R}^d} e^{i\boldsymbol{\omega}^\top(\mathbf{x}-\mathbf{y})}\,p(\boldsymbol{\omega})\,\mathrm{d}\boldsymbol{\omega} = \mathbb{E}_{\boldsymbol{\omega}}\big[e^{i(\boldsymbol{\omega}^\top\mathbf{x}-\boldsymbol{\omega}^\top\mathbf{y})}\big], \tag{S30}$$

where $p(\boldsymbol{\omega})$ is the kernel's spectral density (for the RBF kernel, $p(\boldsymbol{\omega}) = \mathcal{N}(\mathbf{0},\ell^{-2}I_d)$).

Taking the real part and using $e^{i\alpha} = \cos\alpha + i\sin\alpha$ yields

$$k(\mathbf{x},\mathbf{y}) = \mathbb{E}_{\boldsymbol{\omega}}\big[\cos(\boldsymbol{\omega}^\top\mathbf{x})\cos(\boldsymbol{\omega}^\top\mathbf{y}) + \sin(\boldsymbol{\omega}^\top\mathbf{x})\sin(\boldsymbol{\omega}^\top\mathbf{y})\big] \tag{S31}$$

$$= \mathbb{E}_{\boldsymbol{\omega}}\Big[\underbrace{\begin{pmatrix}\cos(\boldsymbol{\omega}^\top\mathbf{x})\\ \sin(\boldsymbol{\omega}^\top\mathbf{x})\end{pmatrix}}_{:=\psi_{\boldsymbol{\omega}}(\mathbf{x})}\cdot\underbrace{\begin{pmatrix}\cos(\boldsymbol{\omega}^\top\mathbf{y})\\ \sin(\boldsymbol{\omega}^\top\mathbf{y})\end{pmatrix}}_{:=\psi_{\boldsymbol{\omega}}(\mathbf{y})}\Big]. \tag{S32}$$

Thus one natural feature map uses both cosine and sine components $\psi_{\boldsymbol{\omega}}(\mathbf{x}) \in \mathbb{R}^2$.

To avoid carrying two trigonometric components per frequency, it is standard to introduce a random phase $b \sim \mathrm{Unif}[0,2\pi]$ and use a single cosine feature. A direct computation shows that

$$\mathbb{E}_{c\sim\mathrm{Unif}[0,2\pi]}\big[2\cos(\boldsymbol{\omega}^\top\mathbf{x}+c)\cos(\boldsymbol{\omega}^\top\mathbf{y}+c)\big] = \cos(\boldsymbol{\omega}^\top\mathbf{x})\cos(\boldsymbol{\omega}^\top\mathbf{y}) + \sin(\boldsymbol{\omega}^\top\mathbf{x})\sin(\boldsymbol{\omega}^\top\mathbf{y}), \tag{S33}$$

because the cross–terms integrate to zero when c is uniform on $[0,2\pi]$. Therefore,

$$k(\mathbf{x},\mathbf{y}) = \mathbb{E}_{\boldsymbol{\omega},c}\big[\cos(\boldsymbol{\omega}^\top\mathbf{x}+c)\,\cos(\boldsymbol{\omega}^\top\mathbf{y}+c)\big], \qquad \boldsymbol{\omega}\sim p(\boldsymbol{\omega}),\ c\sim\mathrm{Unif}[0,2\pi]. \tag{S34}$$

The sine terms are thus "hidden" inside the average over the random phase c, and we can approximate the kernel using the scalar random Fourier features

$$z_r(\mathbf{x}) := \sqrt{\tfrac{2}{m}}\cos(\boldsymbol{\omega}_r^\top\mathbf{x}+c_r), \qquad k(\mathbf{x},\mathbf{y}) \approx \mathbf{z}(\mathbf{x})^\top\mathbf{z}(\mathbf{y}), \tag{S35}$$

with $\{(\boldsymbol{\omega}_r,c_r)\}_{r=1}^m$ drawn i.i.d. from $p(\boldsymbol{\omega})\times\mathrm{Unif}[0,2\pi]$. Drawing i.i.d. pairs $\{(\boldsymbol{\omega}_r,c_r)\}_{r=1}^m$ and defining

$$z_r(\mathbf{x}) := \sqrt{\tfrac{2}{m}}\cos(\boldsymbol{\omega}_r^\top\mathbf{x}+c_r), \qquad \mathbf{z}(\mathbf{x}) := \big(z_1(\mathbf{x}),\dots,z_m(\mathbf{x})\big)^\top, \tag{S36}$$

we obtain the Monte Carlo approximation $k(\mathbf{x},\mathbf{y}) \approx \mathbf{z}(\mathbf{x})^\top\mathbf{z}(\mathbf{y})$.

Applying the augmented Stein operator to each scalar feature yields

$$g_r(\mathbf{x};\boldsymbol{\theta}) := \big(\mathcal{B}_{\boldsymbol{\theta},t_0}z_r\big)(\mathbf{x}), \qquad \mathbf{g}(\mathbf{x};\boldsymbol{\theta}) := \big(g_1(\mathbf{x};\boldsymbol{\theta}),\dots,g_m(\mathbf{x};\boldsymbol{\theta})\big)^\top \in \mathbb{R}^m. \tag{S37}$$

Substituting $k(\mathbf{x},\mathbf{y}) \approx \mathbf{z}(\mathbf{x})^\top \mathbf{z}(\mathbf{y})$ into (S27) and expanding shows that, up to a constant factor, the KSD is approximated by the squared norm of the mean Stein feature,

$$\widehat{\mathrm{KSD}}^2_{\mathrm{RFF}}(\boldsymbol{\theta}) := \left\| \frac{1}{N} \sum_{i=1}^{N} \mathbf{g}(\mathbf{x}_i;\boldsymbol{\theta}) \right\|_2^2, \tag{S38}$$

which has $O(Nm)$ time complexity. In the stationary constant–diffusion case, where $\mathcal{B}_{\boldsymbol{\theta},t_0} = \mathcal{A}^{(D)}_{\boldsymbol{\theta}}$, this reduces to the linear–complexity KSD objective used in Eq. (19) of the main text.

Gradients with respect to $\boldsymbol{\theta}$ only require derivatives of the drift $\mathbf{f}_{\boldsymbol{\theta}}$ (the random features $\{\boldsymbol{\omega}_r, b_r\}$ are fixed once sampled), so (S38) is well suited to mini–batch stochastic optimization in the general non–stationary, state–dependent diffusion setting.

S3. GENE–REGULATORY NETWORK EXAMPLE AND KSD-BASED RECOVERY

In this section, we detail the gene–regulatory network (GRN) example used in the main text (Sec. IV B) and describe how we recover its parameters from unordered steady-state data using the global KSD route.

S3.1. Seven-dimensional stochastic GRN model

We construct a 7-dimensional SDE that couples a three-gene regulatory core to two autonomous "driver" oscillators. The state is

$$\mathbf{x} = (p, b, r, x_1, y_1, x_2, y_2)^\top \in \mathbb{R}^7,$$

where p, b, r are the expression levels of three non-driver genes, and (x_1, y_1) and (x_2, y_2) generate two oscillatory driver signals that modulate the dynamics of b and r.

The four driver coordinates follow two noisy limit cycles with slightly different angular frequencies,

$$\mathrm{d}x_1 = \left(\tfrac{\pi}{100} y_1 + x_1(1 - x_1^2 - y_1^2)\right)\mathrm{d}t + \sqrt{0.0002}\,\mathrm{d}w_1, \tag{S39}$$

$$\mathrm{d}y_1 = \left(-\tfrac{\pi}{100} x_1 + y_1(1 - x_1^2 - y_1^2)\right)\mathrm{d}t + \sqrt{0.0002}\,\mathrm{d}w_2, \tag{S40}$$

$$\mathrm{d}x_2 = \left(\tfrac{\sqrt{2}\,\pi}{100} y_2 + x_2(1 - x_2^2 - y_2^2)\right)\mathrm{d}t + \sqrt{0.0002}\,\mathrm{d}w_3, \tag{S41}$$

$$\mathrm{d}y_2 = \left(-\tfrac{\sqrt{2}\,\pi}{100} x_2 + y_2(1 - x_2^2 - y_2^2)\right)\mathrm{d}t + \sqrt{0.0002}\,\mathrm{d}w_4. \tag{S42}$$

These terms correspond to two noisy Stuart–Landau oscillators with base frequencies $\pi/100$ and $\sqrt{2}\,\pi/100$ and small isotropic diffusion $D = 10^{-4}$ in each coordinate (the SDE is implemented as $\mathrm{d}\mathbf{x} = f(\mathbf{x})\,\mathrm{d}t + \sqrt{2D}\,\mathrm{d}\boldsymbol{w}$, so $\sqrt{2D} = \sqrt{0.0002}$).

The three non-driver genes use the standard saturating nonlinearity

$$\pi = \frac{1}{1+p^2}, \qquad \beta = \frac{1}{1+b^2}, \qquad \rho = \frac{1}{1+r^2}. \tag{S43}$$

The two driver inputs BCR and CD40 are smooth functions of the oscillator phases and the current value of b, in a way that is qualitatively consistent with the phase-dependent modulation observed in B-cell signaling[1]:

$$\mathrm{BCR} = 10\,\left(\sin(\alpha_1)^{30}\right)\beta, \tag{S44}$$

$$\mathrm{CD40} = 5\,\left(\sin(\alpha_2)^{30}\right)\beta. \tag{S45}$$

Here, α_1 and α_2 denote the polar angles of (x_1, y_1) and (x_2, y_2), respectively. The exponent 30 makes the drivers sharply phase-selective while remaining smooth.

We parameterize the regulation of p, b, r by seven nonlinear basis functions of (p, b, r),

$$\boldsymbol{\phi}(p,b,r) := \left(\pi,\ \beta,\ \rho,\ \pi\beta,\ \pi\rho,\ \beta\rho,\ \pi\beta\rho\right)^\top \in \mathbb{R}^7,$$

and a 3×7 interaction matrix W acting on these basis functions. Writing $\boldsymbol{i} = (i_1, i_2, i_3)^\top = W\,\boldsymbol{\phi}$, we obtain

$$\begin{bmatrix} i_1 \\ i_2 \\ i_3 \end{bmatrix} = \begin{bmatrix} 0 & 1 & 1 & 0 & 0 & 0 & 0 \\ 0 & 0 & 0 & 0 & 0 & 0 & 1 \\ 0 & 0 & 1 & 0 & 0 & 0 & 0 \end{bmatrix} \begin{bmatrix} \pi \\ \beta \\ \rho \\ \pi\beta \\ \pi\rho \\ \beta\rho \\ \pi\beta\rho \end{bmatrix}, \tag{S46}$$

which serves as the ground-truth interaction matrix W^*.

The SDEs for (p, b, r) read as follows:

$$\mathrm{d}p = \left(10^{-6} - p + 9\,i_1\right)\mathrm{d}t + \sqrt{0.0002}\,\mathrm{d}w_5, \tag{S47}$$

$$\mathrm{d}b = \left(2 - (1 + \mathrm{BCR})\,b + 100\,i_2\right)\mathrm{d}t + \sqrt{0.0002}\,\mathrm{d}w_6, \tag{S48}$$

$$\mathrm{d}r = \left(0.1 - r + \mathrm{CD40} + 2.6\,i_3\right)\mathrm{d}t + \sqrt{0.0002}\,\mathrm{d}w_7. \tag{S49}$$

Collecting all terms, the full 7-dimensional drift can be written as

$$f_{\boldsymbol{\theta}}(\mathbf{x}) = U(\mathbf{x})\,\boldsymbol{\theta} + v(\mathbf{x}),$$

where $\boldsymbol{\theta} \in \mathbb{R}^{21}$ stacks the entries of W row-wise, $U(\mathbf{x})$ encodes the basis functions for each of p, b, r, and $v(\mathbf{x})$ contains the parameter-free parts (the driver oscillators and constant terms).

Our goal in this example is to infer the 3×7 matrix W (equivalently, the 21-dimensional vector $\boldsymbol{\theta}$) from unordered 7-dimensional snapshots drawn from the stationary distribution of the SDE.

S3.2. Simulation of the data

We simulate the SDE with the true parameter vector

$$\boldsymbol{\theta}^* = (0,1,1,0,0,0,0,\ 0,0,0,0,0,0,1,\ 0,0,1,0,0,0,0)^\top,$$

which implements the interaction matrix W^* above. The diffusion matrix is constant and diagonal,

$$D = 10^{-4} I_7,$$

so the noise amplitude is $\sqrt{2D} = \sqrt{0.0002}$ in each coordinate.

We integrate the SDE using a deterministic fourth-order Runge-Kutta (RK4) step for the drift plus an Euler-Maruyama step for the noise:

$$\mathbf{x}_{t+\Delta t} = \mathbf{x}_t + \frac{\Delta t}{6}(s_1 + 2s_2 + 2s_3 + s_4) + \sqrt{\Delta t}\,\boldsymbol{\eta}_t \sqrt{2D},$$

where s_k are the usual RK4 stages evaluated with the drift $f_{\boldsymbol{\theta}^*}$, and $\boldsymbol{\eta}_t \sim \mathcal{N}(0, I_7)$.

To obtain approximately i.i.d. cross-sectional samples from the stationary distribution, we proceed as follows:

- Initialize a mini-batch of states $x \in \mathbb{R}^{B \times 7}$ with $B = 2048$, sampling all coordinates from a standard normal, and enforcing $p, b, r > 0$ by taking their absolute values.
- Run a burn-in phase of 30,000 time steps with $\Delta t = 10^{-2}$ using the RK4+noise step above.
- After burn-in, continue simulating and record every 10-th step (thinning factor 10) until we have collected $N = 50{,}000$ samples of $\mathbf{x} = (p, b, r, x_1, y_1, x_2, y_2)$.

This procedure yields a dataset $X \in \mathbb{R}^{N \times 7}$ of unordered steady-state samples, which we then use as input to the global KSD route.

S3.3. Global KSD estimator and linear system in θ

Because the drift is affine in $\boldsymbol{\theta}$ and the diffusion matrix is constant, the Stein features arising from our KSD construction are linear in $\boldsymbol{\theta}$. We approximate the Gaussian RBF kernel using $m = 2048$ random Fourier features with bandwidth ℓ chosen by the median heuristic ([2]) on the simulated data. For each random frequency $\boldsymbol{\omega}_r \sim \mathcal{N}(0, \ell^{-2} I_7)$ and phase $c_r \sim \mathrm{Unif}[0, 2\pi]$ we define

$$z_r(\mathbf{x}) = \sqrt{\tfrac{2}{m}} \cos(\boldsymbol{\omega}_r^\top \mathbf{x} + c_r),$$

and apply the diffusion–Stein operator to each scalar feature, obtaining

$$g_r(\mathbf{x}; \boldsymbol{\theta}) = \big(\mathcal{A}_{\boldsymbol{\theta}}^{(D)} z_r\big)(\mathbf{x}), \qquad r = 1, \ldots, m.$$

For our affine-in-parameter GRN drift, each $g_r(\mathbf{x}; \boldsymbol{\theta})$ can be written as

$$g_r(\mathbf{x}; \boldsymbol{\theta}) = a_r(\mathbf{x})^\top \boldsymbol{\theta} + \nu_r(\mathbf{x}),$$

where $a_r(\mathbf{x}) \in \mathbb{R}^{21}$ depends only on the basis functions $\boldsymbol{\phi}(p, b, r)$ and the projection of $\boldsymbol{\omega}_r$ onto (p, b, r), and $\nu_r(\mathbf{x})$ collects all parameter-free terms (including the driver oscillators and the diffusion contribution).

Averaging over the data gives the empirical mean Stein feature

$$\widehat{\boldsymbol{g}}(\boldsymbol{\theta}) := \frac{1}{N} \sum_{i=1}^{N} \mathbf{g}(\mathbf{x}_i; \boldsymbol{\theta}) = A_{\mathrm{global}}\, \boldsymbol{\theta} + \mathbf{b}_{\mathrm{global}},$$

where $\mathbf{g}(\mathbf{x}; \boldsymbol{\theta}) = (g_1(\mathbf{x}; \boldsymbol{\theta}), \ldots, g_m(\mathbf{x}; \boldsymbol{\theta}))^\top \in \mathbb{R}^m$ and

$$A_{\mathrm{global}} \in \mathbb{R}^{m \times 21}, \qquad \mathbf{b}_{\mathrm{global}} \in \mathbb{R}^m$$

are computed in closed form by batched accumulation over the dataset (batch is used only when sample size is too large). The linear-time RFF approximation to the KSD is then

$$\widehat{\mathrm{KSD}}^2_{\mathrm{RFF}}(\boldsymbol{\theta}) = \big\| \widehat{\boldsymbol{g}}(\boldsymbol{\theta}) \big\|_2^2 = \| A_{\mathrm{global}} \boldsymbol{\theta} + \mathbf{b}_{\mathrm{global}} \|_2^2.$$

Minimizing this quadratic objective is equivalent to solving a regularized linear system. We use Tikhonov regularization with $\lambda = 10^{-6}$ and calculate the $\widehat{\boldsymbol{\theta}} \in \mathbb{R}^{21}$.

Across 50 independent simulation and estimation runs (different random seeds and data draws), the recovered interaction matrices $\widehat{W}$ closely match W^* for most entries, with one coefficient systematically deviating. As discussed in the main text, this misestimate entry lies in a weakly constrained direction of parameter space predicted by our Gram/Hessian analysis, and the corresponding SDE trajectories in (p, b, r) remain practically indistinguishable from those generated by the true parameters.

[1] M. R. Martínez, A. Corradin, U. Klein, M. J. Álvarez, G. M. Toffolo, B. di Camillo, A. Califano, and G. A. Stolovitzky, Proceedings of the National Academy of Sciences **109**, 2672 (2012).
[2] D. Garreau, W. Jitkrittum, and M. Kanagawa, arXiv preprint arXiv:1707.07269 (2017).
